# Genomic and Epigenomic Features of Glioblastoma Multiforme and its Biomarkers

**DOI:** 10.1155/2022/4022960

**Published:** 2022-09-21

**Authors:** Sarmad Sheraz Jadoon, Umair Ilyas, Hajra Zafar, Ana Cláudia Paiva-Santos, Saifullah Khan, Saeed Ahmad Khan, Tanzeel Ahmed, Yasir Rasool, Reem Altaf, Faisal Raza, Muhammad Abbas

**Affiliations:** ^1^State Key Laboratory of Esophageal Cancer Prevention & Treatment, School of Pharmaceutical Sciences, Zhengzhou University, China; ^2^Department of Pharmacy Practice, Riphah Institute of Pharmaceutical Sciences, Riphah International University, Islamabad, Pakistan; ^3^School of Pharmacy, Shanghai Jiao Tong University, Shanghai 200240, China; ^4^Department of Pharmaceutical Technology, Faculty of Pharmacy of the University of Coimbra, University of Coimbra, Azinhaga Sta. Comba, 3000-548 Coimbra, Portugal; ^5^LAQV, REQUIMTE, Department of Pharmaceutical Technology, Faculty of Pharmacy of the University of Coimbra, University of Coimbra, Azinhaga Sta. Comba, 3000-548 Coimbra, Portugal; ^6^Institute of Biotechnology and Microbiology, Bacha Khan University, Charsadda, Pakistan; ^7^Department of Pharmacy, Kohat University of Science and Technology, Kohat, Pakistan; ^8^Bolan Medical Complex Hospital, Quetta, Pakistan; ^9^Akhtar Saeed College of Pharmacy, Bahria Golf City, Rawalpindi, Pakistan; ^10^Department of Pharmacy, Iqra University-Islamabad Campus, Islamabad, Pakistan

## Abstract

Glioblastoma multiforme is a serious and life-threatening tumor of central nervous system, characterized by aggressive behavior, poor prognosis, and low survival rate. Despite of the availability of aggressive antitumor therapeutic regimen for glioblastoma (radiotherapy followed by chemotherapeutic dose), recovery rate, and patients' survival ratio is attributed to the lack of selectivity of therapeutic drugs and less advancement in cancer therapeutics over last decade. Moreover, tools employed in conventional diagnosis of glioblastoma are more invasive and painful, making the process excruciating for the patients. These challenges urge for the need of novel biomarkers for diagnosis, prognosis, and prediction purpose with less invasiveness and more patient compliance. This article will explore the genetic biomarkers isocitrate dehydrogenase mutation, MGMT mutations, and EGFR that can be deployed as an analytical tool in diagnosis of disease and prognosis of a therapeutic course. The review also highlights the importance of employing novel microRNAs as prognostic biomarkers. Recent clinical advancements to treat GBM and to prevent relapse of the disease are also discussed in this article in the hope of finding a robust and effective method to treat GBM.

## 1. Introduction

Glioblastoma multiforme (GBM) is the grade IV malignant tumor of the neural stem cells, which is specified on the basis of its hostile nature that emerges from histopathological differences and genomic variations among the patients [1–3]. GBM is the most frequently occurring primary brain tumor in older people. It comprises almost 50% of neuroglial tumors and 12-15% of intracranial tumors. Patients suffering from GBM experiences poor diagnosis and have less chances of survival (2-5%) [4, 5]. The ultimate treatment of GBM is the surgical removal of tumor, accompanied by chemotherapy and radiotherapy. Even after the aggressive treatment, relapse may occur in some patients due to the molecular diversity of disease. To overcome this, a large number of molecular, diagnostic, predictive, and prognostic biomarkers have been identified by researchers. These biomarkers are collected by taking fluids from the patient's body or biopsy and are examined by polymerase chain reaction along with other investigation techniques [6]. Some of these biomarkers include mutation of isocitrate dehydrogenase, methylation of O6-methylguanine-DNA methyltransferase, amplification of epidermal growth factor receptor vIII, and omission of 1p19q. Some microRNAs especially miR-10b and miR-21 are also used as prognostic biomarkers. These biomarkers play an integral role in the development of anti-GBM therapies such as vaccines, drug conjugates utilizing antibodies, and growth factor receptor's inhibitors. The most advanced biomarkers are the inhibitors of immune checkpoints. The investigation of current and novel biomarkers leads to the successful treatment of GBM [7].

## 2. Genetic Hallmarks of GBM (Glioblastoma Multiforme)

**Figure 1:**
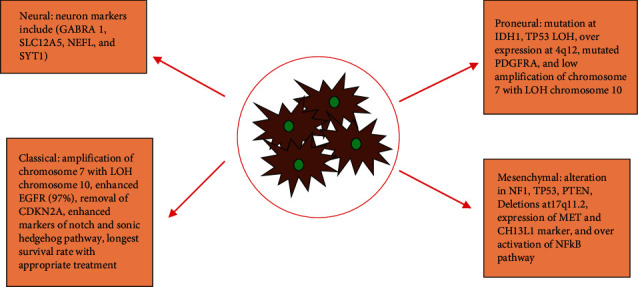
Molecular biomarkers of GBM associated with Verhaak subtype classification of GBM.

Types and molecular subtypes: GBM are of two main types, primary and secondary GBM. Primary glioblastoma occurs without any pre-existing disease and is commonly developed in older people. It is asymptomatic before being malignant but becomes symptomatic right after attaining malignancy. Primary glioblastoma accounts for 90% of total GBM cases around the world. It has poor diagnosis as compared to secondary GBM. This subclass of GBM is characterized by mutation in phosphatase and tension homolog (PTEN), loss of heterozygosity (LOH) 10q, and epidermal growth factor receptor (EGFR) amplification [8]. Secondary GBM accounts for the 5% of total GBM worldly cases, mainly affecting young people. The main biomarker that contributes towards the progression of secondary GBM is the alterations in tumor protein 53. On the basis of Verhaak classification, GBM consist of some distinct subclasses named as proneural, classical, neural, and mesenchymal subclass (Figure 1) [9].

In proneural subclass, IDHq alteration and mutation in platelet-derived growth factor receptor alpha (PDGFRA) is observed, whereas neural GBM subtype is characterized by over expression of EGFR, removal of CDKN2A, and alteration in TP53. Over expression of MET genes and chitinase 3-like genes are seen in mesenchymal subclass [9]. The last amplification of EGFR and LOH is observed in classical subtypes [10]. The following diagram summarizes the GBM subtypes along with their genetic aberrations.

Neural, mesenchymal, and classical subtypes are similar on the basis of diagnosis, whereas proneural subtype shows differential prognosis and commonly found in younger people with high survival rate [9]. The reason is that proneural subclass is majorly associated with secondary glioblastomas (85%) having IDH1 gene mutations, whereas other three subclasses are linked to primary glioblastomas showing less (5%) or no alteration in IDHI gene [11].

## 3. Epigenetic Alterations Associated with Glioblastoma Multiforme

A variety of methods and tests are utilized for collecting information about different classes of genomic biomarkers in GBM. The accuracy of these tests and methods will eventually lead to the correct diagnosis and treatment of the disease [12].

### 3.1. Methylation Signature in GBM

The main molecular biomarkers in the GBM are PDGFRA, IDH, O^6^-methylguanine-DNA methyltransferase (MGMT), EGFR, p16INK4A, NF1, and VEGF. The ultimate goal of studying these genomic biomarkers is to establish the possible methods for the reversal of tumor progression and disease treatment [10].

The following Table 1 explains the summarized form of genomic biomarkers of glioblastoma multiforme along with their importance.

#### 3.1.1. Alteration and Intensification of EGFR, IGFR-1, and FGFR1 (RTK Signaling Pathway)

Enhancement or alterations of PDGFRA, EGFR, insulin-like growth factor receptor (IGFR-1), and basic fibroblast growth factor receptor 1 (FGFR1) accounts for 80% of the primary glioblastoma [13]. These proteins are linked together structurally and generate an intricate network of signals that controls many cellular mechanisms. RTK signaling pathway uses two major pathways; PI3K/AKT/mTOR pathway that regulates cell cycle and inhibit programmed cell death and RAS/RAF/MAPK pathway that is mainly involved in cellular movement, multiplication, and segregation [13]. Tumor suppressor gene (PTEN) negatively regulates the PI3k pathway [14]. Removal of PTEN gene occurring in almost 36% of GBM and it causes over expression of P13K pathway that leads to the development of chemoresistance [15].

Local amplification, dislocation, and alteration of endothelial growth factor receptor is the most commonly occurring genetic aberrations in GBM. It accounts for 57% of the total GBM [16]. Augmentation of EGFR is mainly present in classical subtype of GBM [17].

**Figure 2:**
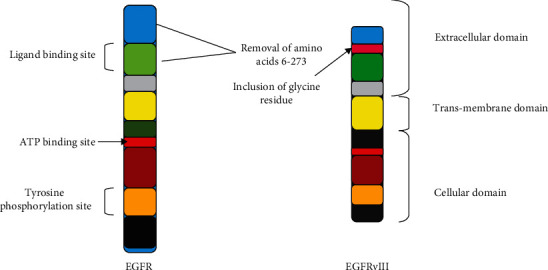
Endothelial growth factor receptor and its mutated form.

EGFR gene regulates the EGFR and generates a genetic code particularly for a tyrosine kinase receptor. EGFR is mutated by the alteration of histone protein present on its gene enhancer, positioned at chromosome 7p12 [18]. This alteration causes reduction of exon 2 and 7 and leads to the formation of EGFRvIII that lacks extracellular ligand binding site (Figure 2).

Amplification and alteration of EGFR are plentiful in necrotic GBM samples so they are considered as a prognostic biomarker of GBM [19].

EGFR regulates the ligand-dependent signaling, whereas EGFRvIII is not able to bind itself with a ligand and shows less activity as compared to EGFR. As a result, EGFR can cross-phosphorylate with EGFRvIII in the presence of EGF [20, 21]. Patients with no EGFRvIII mutation live longer (survival rate 1.4 years) as compared to those having EGFRvIII mutation (survival rate 0.8 years) [18].

Latest research suggests that the amplification of EGFR as a prognostic biomarker is not compatible to some extent. A lot of reports are available that indicate its negative impact on overall survival rate of patients, some reports show positive effect on OS rate and some shows no relation at all [22–25]. Mutation and amplification of EGFR in GBM may limit the efficiency of EGFR targeting drugs such as small molecule inhibitors and immunotherapy [15, 26]. Although rate of EGFR gene amplification is high but due to these contradictory results, clinical trials are not performed on EGFR inhibitors (erlotinib and gefitinib) [27, 28].

#### 3.1.2. Methylation of MGMT (O^6^-Methylguanine-DNA Methyltransferase) Promoter

**Figure 3:**
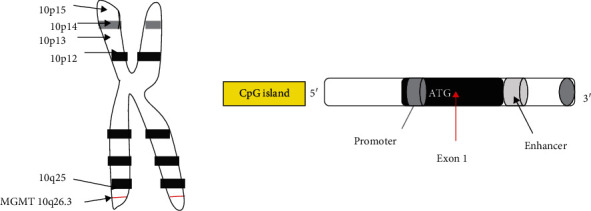
Location of MGMT gene on chromosome and CpG island in MGMT gene.

MGMT (O^6^-methylguanine-DNA methyltransferase) is known as a DNA repair enzyme because it shifts the methyl group placed at O^6^ position of guanine to its cystine parts and conserves the destroyed guanine nucleotides. The site of location of MGMT gene is 10q26.3 on chromosome with a length of 300,437 bp [29, 30], (Figure 3). Epigenetic alteration controls the expression of MGMT gene.

It prevents necrosis, G: C → A: T [31] genetic alterations and carcinogenesis induced by alkylating agent. MGMT gene eliminates the alkylating agent by encoding DNA repair protein. As a result, chemoresistance occurs. Methylation of CpG site of MGMT promoter diminishes MGMT expression, thus, reduces repairement of DNA and allows more penetration of alkyl groups [32–34]. As a result, alkylating agents become more powerful in patients with hypermethylation of MGMT promoter. Methylation of MGMT promoter is more commonly seen in secondary glioblastomas as compared to primary glioblastomas [35, 36]. Recent research suggests that methylation of MGMT promoter enhances the overall survival (OS) and progression-free survival (PFS) in patients having alkylating agents [31, 37]. Thus, it acts as a prognostic biomarker of GBM and estimates the overall responsiveness of patient towards alkylating agent.

In addition, the two latest clinical trials, the RTOG 0525 and the Nordic trial (NOA-08), suggest that methylation of MGMT promoter also helps to distinguish the response of older GBM patients for chemotherapy with alkylating agent and radiotherapy. It has been observed that the patients with tumor with methylated MGMT promoter have better survival rate when treated with combination therapy (radiation therapy along with chemotherapy with TMZ) as compared to those having unmethylated MGMT promoter tumor [38–40]. Therefore, testing of MGMT promoter methylation is done in older GBM patient before the initiation of any treatment. Methylation of MGMT promoter also improves the PFS and OS in patients having intermittent GBM, hence, it is considered as a predictive biomarker for the selection of treatment strategy.

#### 3.1.3. Isocitrate Dehydrogenase

**Figure 4:**
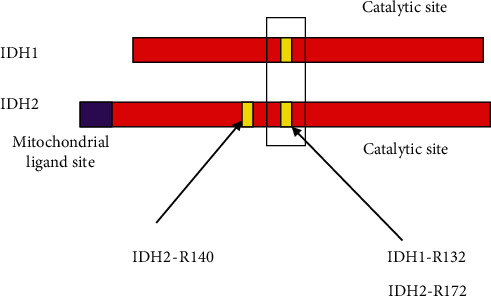
Site of mutation in IDH1 gene is R-132 and in IDH2 gene is R-172.

Isocitrate dehydrogenase comprises of two metabolic enzymes: Isocitrate dehydrogenase 1 (found in cytosol and peroxisome) and Isocitrate dehydrogenase 2 (found in mitochondrial site) that are programmed by two IDH genes, IDH1, and IDH2. Enzymes of IDH are mainly responsible for catalyzing oxidative carboxylation of isocitrate to produce alpha-ketoglutarate. As a result of this catalysis, NADPH is produced in the kreb or citric acid cycle [41, 42]. Alteration in IDH genes promote the conversion of alpha-ketoglutarate in to 2-hydroxyglutarate which is an oncometabolite [43, 44]. 2-HG causes the hyper methylation of DNA promoter known as glioma-CpG island methylator phenotype (G-CIMP) and promotes tumorigenesis [45]. In GBM, IDH gene mutation is commonly found at the 132 chromosomal residue in IDH1 and at 172 residue in IDH2 (Figure 4).

IDH protein mutation is uncommon in primary GBM (5%) and it is mainly present in secondary GBM (73-85%) [46, 47]. Recent research suggests that mutation in IDH gene also leads to the development of other genomic deformities like removal of 1p/19q chromosome or alteration in TP53 gene and is found to be present along with amplification of EGFR and removal of chromosome 10 [11]. Furthermore, it causes mutation in other genes such as mTOR gene and ATM gene that is closely linked with the progression of GBM [48].

As in GBM patients, IDH protein mutation is associated with improved PFS (progression-free survival) and OS (overall survival) rate so this aberration is considered as one of the most important molecular prognostic biomarker of GBM [49]. It also encourages the production of new chemotherapeutic agents that inhibits IDH protein mutation and shows highest clinical efficiency [50].

#### 3.1.4. CD44

CD44 is a transmembrane glycoprotein molecule that is involved in cell division, programed cell death, and new blood vessel formation.

CD44 expression plays significant role in invasion and metastasis of glioma cells and higher levels of CD44 in patients act as a glioblastoma cancer stem cell marker. Cancer stem cells are glioma initiating cells (GICs) or progenitor cells that can initiate proliferation of the cells at secondary sites after metastasis of primary glioma cells [51].

**Figure 5:**
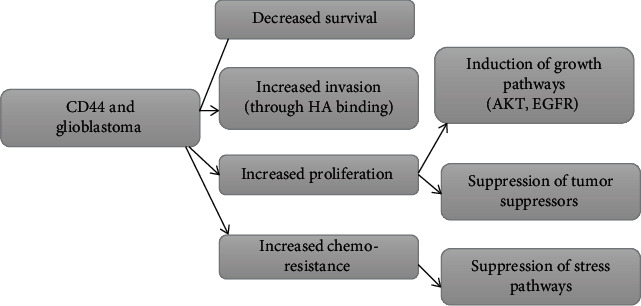
CD44 role in glioblastoma.

Glioblastoma cancer cells show increased resistance to radiation and overexpression of genes Sox2, Nanog, Id1, and Oct4. CD44 induces upregulation of genes that are involved in tumor modulation. This modulation is initiated by binding of osteopontin ligand with the extracellular part of CD44. CD44 also functions as a receptor site for hyaluronic acid (HA) in glioblastoma patients. HA binding of CD44 results in stimulation of downstream cascade of pathways, activation of growth pathways, and under expression of tumor suppressor mechanism. In almost 60% of the patients with glioblastoma, CD44-associated EGFR receptor upregulation is involved that result in increased adhesion of tumor cells to cellular base lines and invasion to neighboring normal cells. CD44 also upregulates the mediators (Akt and Erk 1/2 kinase) of EGF pathways (Figure 5) [52].

CD44 expression is associated with increased proliferation of the cells, which in turn is due to stimulation of growth pathways (AKT and EGFR), by suppression of tumor suppressor genes, increased resistance to chemotherapy, and invasion of tumor cells to normal healthy cells.

There exists a relationship between CD44 overexpression and tumor grade. CD44 is overexpressed in mesenchymal subtype of glioblastoma. RFX1 is a regulatory transcription factor that downregulates the proto-oncogene. RFX1 performs its function by binding with ectodomain of CD44 and downregulates CD44 expression resulting in reduced phosphorylation of Erk and Akt, thus have negative effect on glioblastoma progression and invasion [53].

CD44 is also associated with downregulation of Lats1/2 apoptotic pathway that results in increased tumor metastasis and resistance to drug therapy. CD44 induces resistance to chemotherapy most probably by suppressing apoptotic response. A trial showed that depletion of CD44 make glioblastoma cells susceptible to chemotherapy and overexpression of CD44 make more colonies of glioma cells even after chemotherapeutic dose.

**Figure 6:**
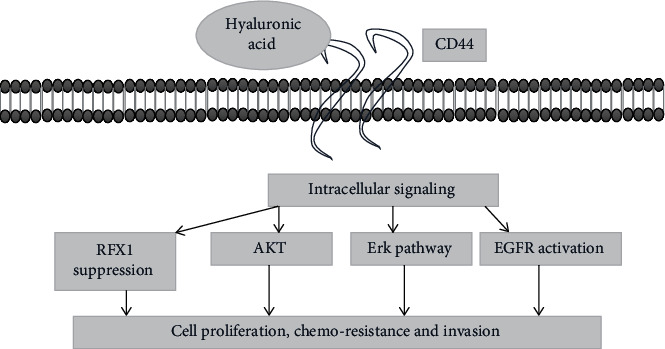
Mechanism of CD44 induced tumor progression.

One of the therapeutic approaches to treat overexpressed CD44 tumor cells can be the downregulation of CD44 molecule by targeting with antisense vector or monoclonal antibodies. Treatment with monoclonal antibodies also has a positive impact on the sensitivity to chemotherapeutic agents resulting in a decrease invasion and metastasis of tumor cells (Figure 6) [54].

Binding of stimulants to CD44 results in activation of intracellular cascade, activation of growth regulation pathways, and suppression of tumor suppressor pathways that results in increased cell division, resistance against chemotherapeutic agents, and invasion of glioma cells to normal healthy cells.

#### 3.1.5. Tumor Protein Tp53 Inactivation

**Figure 7:**
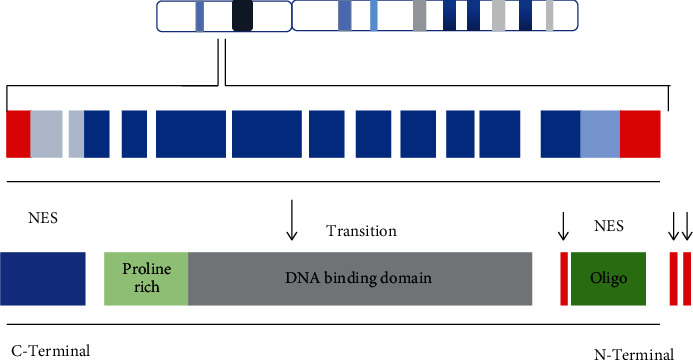
Mapping of Tp53 on chromosome 17p13. Structure and location of chromosomes and the distribution of protein domains on chromosomal site.

Tumor protein p53 is a transcription factor, a tumor suppressor that is located on chromosome 17p13.1 encoding for 393 amino acids (Figure 7).

Tp53 has domains like DNA binding domain, transactivation domain, proline rich site, and oligo domain. It has19, 149 nitrogenous base pairs with 11 axons.

Tp53 in glioblastoma: under normal circumstances, Tp53 suppresses tumor activity by modulating the expression of genes involved in cell cycle, division and differentiation of the cells, and apoptosis. Activity of p53 is controlled and well-checked by MDM4 and MDM2. In both p53 and MDM2, MDM4 functions through negative feedback mechanism. Though mutation in IDH1 and MGMT are better markers for glioblastoma than p53, however p53 can act as genetic marker for glioblastoma [55].

According to cancer genome atlas (TCGA, 2013), deregulation of ARF-MDM2-p53 pathway occurs in glioblastoma. Any mutation in Tp53 is linked with progression of glioblastoma. ARF facilitates the degradation of MDM2 and upregulates expression of tissue inhibitor of metalloproteinase-3 (TIMP3). Its inactivation leads to increased proliferation of the cancer cells, invasion, and immortality of the tumor cells. In almost 60% of glioblastoma cases, p53 inactivation is caused by the deletion of CDKN2A/ARF locus. ARF deletion leads to increased expression of tectonic family member 1 (TCTN1) protein that results in increased promotion of glioblastoma [17].

Over expression of MDM2 and MDM4 results in inactivation of p53 that in turn results in the loss of p53 activities, a reduction in DNA repair, and decreased cell death and cell differentiation. MDM4-induced p53 inactivation is more common in classic glioblastoma. Tp53 mutations are present in both types of glioblastoma, primary and secondary glioblastoma.

Genetic aberration associated with TP53/MDM2/p14ARF pathway accounts for 87% of cases of glioblastoma, over expression of MDM2 induces GBM in 14% patients, 49% GBM roots out from p14ARF homozygous deletion, and Tp53 mutation results in 35% of glioblastoma cases [56].

Therapeutic approaches for p53 inactivation-induced glioblastoma: though variety of genetic therapies have been employed for treating glioblastoma, but these treatment provides inadequacy due to resistance to chemotherapeutic agents, loss of selectivity, and recurrence of the disease. Inactivation of p53 in glioblastoma provides a way to specific and selective treatment of glioblastoma. Researches have been made for years to design oncolytic viruses to treat GBM. Approaches are being employed to treat p53 inactivation-induced glioblastoma include interference with the interaction of p53 and MDM2 to sensitize tumor cells against chemotherapy.

Another approach that can be employed is p53 plasmid-mediated transfection that inhibits and arrests the cell cycle in G1 or Go phase is preventing the entry of cells into synthesis phase, thus, no replication of DNA and no new cells formation occurs. Its example oral nutlin-3, a promising molecule when tested on animal models, showed striking results with increased survival rate in mice. Clinical trials for its use in humans are under research.

#### 3.1.6. Loss of Heterozygosity of Chromosome 10

Loss of Heterozygosity of chromosome is normally present in all types of GBM. It promotes the uncontrolled division of tumorous cells by affecting the tumor suppressor genes [57]. In glioblastoma multiforme, LOH mainly affect 10, 9p, 19q, 22, and 17p; whereas, LOH19q and LOH1p chromosomal regions are considered as predictors of oligodendrocyte neoplasms [58]. LOH of chromosomal region 10q more particularly 10q23 comprises a major portion of GBM (70% of all the types) but mainly present in primary glioblastoma multiforme [59].

On the basis of results obtained from different studies, LOH chromosome 10 is considered as a diagnostic biomarker for primary and secondary GBM: whereas, LOH10q25-qter in particular is used only for the diagnosis of secondary GBM [60]. The tumor suppressor genes that are affected by LOH10q are TP53, PTEN, and NF1. PTEN gene is responsible for inhibiting PIP3 that reduces cellular multiplication and causes programmed cell death. PTEN gene after being affected by LOH10q modulates the PI3k pathway and promotes cellular multiplication [13].

#### 3.1.7. Circulating Tumor Cells

Tumor cells that detach from their primary attachment site, move to the adjacent cells, and travel through the bloodstream to reach distant areas where they divide and survive, thus forming new colony (other than primary attachment site) are called circulating tumor cells. In glioblastoma, circulating tumor cells (CTCs) spread the disease to the neighboring cells. Thomas Ashworth for the first time in 1869, witness the presence of CTCs in blood stream [61].

The National Comprehensive Cancer Network made grading of biomarkers based on the extent of their role in diagnosis and prognosis. Circulating tumor cells either they are alone cells, extracellular vesicles, or in the form of circulating clusters are graded as promising biomarkers in glioblastoma [62].

**Figure 8:**
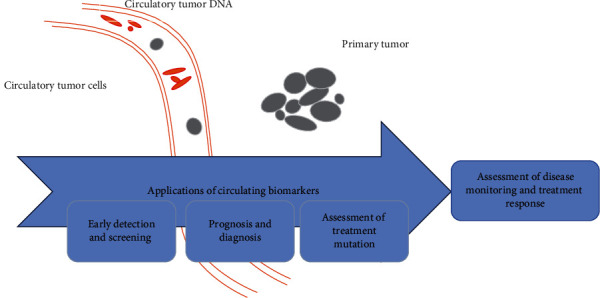
Tumor shed their cells (circulating tumor DNA, extracellular vesicles, and circulating tumor DNA) into the blood stream. These markers can be used in liquid biopsy for management of the disease (disease diagnosis, screening, and prognosis).

CTCs serve as prognostic markers for glioblastoma and have a prevalence of greater than 75% in GBM. CTCs have direct correlation with tumor progression, recurrence, and type of GBM. Levels of circulating tumor cells decrease after treatment as compared to their level prior to the treatment indicating their role as a prognostic marker [63]. CTCs can be analyzed by telomerase assay. CTCs are present in the blood of cancer patients, where they can be easily detected in blood sample through liquid biopsy or blood test. The employment of CTCs as a diagnostic marker for glioblastoma is of significant importance as it eliminates the need of conventional tumor biopsy processes. Conventional biopsy processes are more invasive in nature with only one snapshot. While use of CTCs “liquid biopsy” provides information about all solid tumors and are less invasive and less painful even after several repeats [64], (Figure 8). Recent studies highlight the importance of CTCs in genetic profiling of cancer patients to drug sensitivity.

As a diagnostic tool, presence of CTCs in blood indicates tumor and an increase in CTCs with the passage of time shows disease progression. Number of CTCs in blood sample reflects therapy effectiveness. After chemotherapy, CTCS serve as prognostic marker, unchanged number of CTCs after chemotherapy shows therapy resistance, and a decreased CTC count is an indication of success of therapy.  Table 2 represents the advantages and disadvantages associated with CTCs.

*(1) Glioma-Derived Exosomes*. Extracellular vehicles (EVs), containing protein, lipids, DNA, mRNA, and noncoding RNAs, bud from the cell surface and are involved in the transfer of biomolecules bound to membrane to the neighboring cells or extracellular fluids. A range of characteristic pathological features of EVs plays a pivotal role in the malignant progression of GBM. As nanoscale vesicles with the natural ability to cross the blood-brain barrier (BBB) [65], tumor-derived exosomes are key mediators in mediating intercellular communication between metastatic cancer cells and brain stromal cells to complete brain metastasis colonization [66, 67], as well as inducing epithelial mesenchymal transition (EMT) in neoplastic epithelial cells and conferring them intravasation and migration ability [65, 67]. In addition, tumor-derived exosomes contribute to premetastatic milieu creation, tumor development, progression, immune evasion, angiogenesis, antiapoptotic signaling, and treatment resistance throughout their bioactive cargo [68]. They can be used to modify the microenvironment of the primary tumor and make targeted organs suitable for tumor progression. Examples include promoting ECM remodeling, facilitating the cancer-associated phenotype transformation of fibroblasts, and increasing the neural distribution of the tumor microenvironment [69]. Among these, it is worth mentioning that tumor-derived exosomes facilitate the formation of an immunosuppressive tumor microenvironment, where they can assist tumor cells to evade immunity by reducing immunogenicity, inducing suppressor cells, modulating antigen presentation, and secreting immunosuppressive factors [70]. Furthermore, the toxic potential of GBM-derived exosomes to primary neurons is one of the important factors explaining the perineural edema and cognitive decline in GBM patients [71]. There are four types of EVs, exosomes, membrane particles, microvesicles, and apoptotic vesicles. The role of exosomes as therapeutic target and drug delivery system in the diagnosis and progression of glioma has been well explored. In the diagnosis of gliomas, the mRNA, miRNA, protein, and DNA are beneficial. The disease detection and progression can be performed by tumor-specific RNA in the serum exosomes. The mutant 1DH1 transcript was detected in exosomes isolated form CSF of glioma patient. Moreover, the p65 genes and the dynamin 3 (DNM3) genes were found to be upregulated in the exosomes derived from patient shaving primary and recurrent GBM providing the evidence of these specific transcripts as potential diagnostic marker for GBM. A high level of miR-21, miR-222, and miR-124-3p were observed in the serum exosomes of high grade glioma patients. The miR-574-3p, miR-320, and RNU6-1 levels were also isolated from serum exosome of GBM patients and healthy ones with RNU6-1 having diagnostic potential of GBM. miR-301a levels provide the pathological status of the glioma and its levels are markedly increased in the isolated exosomes form GBM individuals [72]. The circRNA has also shown important role in the diagnosis of GBM derived from the exosomes. These include circSMARCA5 and circHIRK2 [73]. In protein content, EGFR, EGFRVIII, podoplanin, and IDH1 showed protein expressed in the exosomes. About 133 protein in the exosomes from glioblastomas were detected in a study suggesting these proteins can serve as markers to create techniques to diagnose disease [72, 74]. The mutations in gene IDH1 in the exosomes of patient suggested the role of DNA detection in GBM. The therapeutic potential of cell-derived exosomes have also been explored. The human umbilical cord-derived mesenchymal stem cells exosomes have partial antitumor activity. Exosomes derived from NK cell have also shown antitumor role. The chemical modification and genetic engineering of exosomes could improve the therapy against GBM. The exosomes have also role in drug and gene delivery due to its inability to trigger immune responses and are considered as innovative delivery system. The transfer of curcumin and doxorubicin through exosomes has also been studied. The miRNA delivery of exosomes is also a promising GBM therapy target [72].

### 3.2. microRNA

microRNAs are noncoding, short length RNA molecules that show their effect in the development and spread of tumor. miRNAs play their role through modulation of tumor suppression and activation genes. Many studies have focused in miRNA-targeted therapy to be utilized in cancer treatment. The miRNA mimetic and antimiRNA agents have been developed for this purpose. The differential expression analysis have also helped in this regard in the identification of miRNAs having potential role in GBM development [75]. In one study, a Connectivity Map (CMap) method was employed to determine the miRNA-based therapeutic agents for GBM treatment. About 10 differentially expressed miRNA were identified sowing association with eight GBM-associated genes. These genes include RB1, PRKCB, CALM3, CDK6, CAMK2G, NRAS, PDGFRA, and CAMK2B. These genes may participate in the development of GBM [76]. In another study, the association of malignant types tumors with the neurological disorders were studied, identifying the association of Alzheimer's disease (AD) with that of glioblastoma. The study identified potential specific miRNAs that have shown to be deregulated in both diseases. In GBM, miR-7 and miR-93 have shown downregulation in glioblastoma along with miR-128 and miR-139. In AD, the downregulation of miR-29c was observed which can serve as a biomarker or therapeutic agent in AD. The downregulation of this microRNA has also been observed in GBM [77]. This provides the important function of miRNA as potential oncogenes and tumor suppressors that can serve as prognostic biomarkers and therapeutic targets in GBM [75].  Table 3 shows the summary of miRNAs employed as biomarkers.

miRNA's samples are gathered from body fluids (blood, CSF, or urine) to employ these samples as markers of GBM. This technique is a less invasive approach towards gathering biomarkers. miRNA provides more than 90% accurate results when employed as diagnostic biomarker [78].

miR-21: miR-21 expresses itself in GBM where it acts as a modulator of tumor suppressor genes, RECK, FasL, and PDCD4. A decrease in miR-21 or its inhibition increases the rate of cell death and decreases division of the tumor cells. Moreover, miR-1 plays its role in upregulation of cancer stem cells [79].

miR-10b: miR-10b is highly expressed in most subtypes of GBM. miR-10b acts as a valuable prognostic biomarker of glioblastoma. miR-10b induces tumor by splicing MBNL and RSRC1 genes, by inhibition of Bcl-2, and causes increased cell proliferation [80]. It induces resistance to chemotherapy by activating AKT pathway [81].

miR-15b: mir-15b performs its role as prognostic biomarker by halting cell cycle and by inhibiting proliferation of the cells.

miR-137: miR-137 induces suppressor effects same like miR-15b. It negatively regulates target gene (GLIPR-1) as miR-137 promoter is excessively methylated in glioblastoma. Other miRNAs that play their role in modulating chemoresistance include miR-127, miR-603, miR-181d, and miR-648. To effectively use miRNA as a therapeutic choice, nanoparticles and liposomes are being employed in past decades [82].

### 3.3. lnRNA

During transcription of human genome about less than 3% of all transcribed genes are protein coding, with 70% of human genome transcription majority of the noncoding transcripts of various sizes are produced. These are the noncoding RNAs (ncRNA) and are categorized into two main categories the long ncRNAs and the small ncRNAs. The lncRNAs have a wide phenotypic impact having essential role in regulation of transcription, subcellular localization, and epigenetic remodeling. Recently, evidences have suggested prognostic and therapeutic implications of lncRNAs in GBM and is an evolving field. Several lncRNAs have shown regulation of glioma tumors originating with tumor initiation and progression [84, 85].  Table 4 enlists the lncRNAs employed as therapeutic markers.

### 3.4. circRNA

circRNA is formed through back-splicing from premiRNA as a result of protein-coding genes. These are noncoding RNA with covalently closed RNA molecule. Evidences have shown the involvement of circRNA in the regulation of gene expression. Because of their closed loop structure, they have longer half lines and are naturally resistant to degradation. However, circRNAs are mostly expressed at low levels. Their role in tumor progression has also been studied and have shown to be directly translated into proteins regulating protein functions. The dysregulation of circRNA expression levels is also associated with several pathological conditions including gliomas and GBM. One of the circRNA identified include the circSMARCA5. It is a tumor suppressor circRNA. The expression of circSMARCA5 is downregulated in GBM tissue. The mutation of GAUGAA RNA motif that is involved in its interaction with SRSF1 causes a significant decrease in the binding between SRSF1 and circSMARCA5 leading to decreased GBM cell migration and angiogenic potential [111]. Another important circRNA identified is the circLGMN, which is significantly upregulated in high grade glioma. This circRNA have shown regulation of mammalian legumain (LGMN) promoting GBM malignancy [73]. However, further studies are required on circRNAs as only few circRNAs have been studied.

## 4. Imaging of Biomarkers

To date, there is no approved imaging biomarker there, but researches are in process to develop advanced functional techniques for imaging biomarkers. These techniques include weighted magnetic resonance imaging, positron emission tomography, MR spectroscopy, and dynamic susceptibility weighted-contrast enhanced perfusion imaging [112].

These techniques will help in deciding personalized treatment for patients after successful molecular diagnosis of the disease. In a study, the level of 2-hydroxy glutarate when checked by mass spectroscopy showed an increase in its concentration, indicating direct correlation with IDH1 and IDH2 that in turn indicates the presence of the tumor [113].

The biomarkers for EGFR amplification may include increased cerebral blood volume, lower values of ADC, and a decrease in the ratio of necrotic tissues to contrast enhancing tissue. Positron emission tomography can be employed as a potential tool for imaging biomarkers. One of the advancement being made in the PET technology is F-FDG ligand use, but it has limitations to not detect small tumors because of an increased glucose intake by brain tumor cells. Other ligands that are under assessment for last decade include amino acids having radioactive materials, methionine having radioactive carbon at position 11 (11C-MET), 3,4-dihydroxy-6-18F-fluoro- L-phenylalanine (^18^F-FDOPA), 18F-flouroethyl tyrosine (^18^F-FET), and 18-F fluoromisonidazole.

11C-MET was found effective in elevating the survival rates in glioma patients when trialed by Baek et al. F-FDOPA finds its role as a differentiating biomarker as it can differentiate between low and high grade glioma [112].

### 4.1. Summary

Genes participate in signaling pathways of the cells and are associated with division of the cells, programmed cell deaths, and blood vessel formation for newly formed cells. Hsu et al. mentioned ten genes associated with GBM (glioblastoma multiform) in his work. These genes act as potential biomarkers for identifying glioma in the patients' prognosis and determine the molecular subtype of glioma in individuals [114].

Genetic changes linked with tumor include alterations in isocratic dehydrogenase (IDH), changes in epidermal growth factor receptor, platelet-derived growth factor receptor changes, aberrant epigenetic changes, and loss of heterozygosity of 1p/19q [49] (Table 5).

## 5. Conclusion and Future Directions

Glioblastoma is a devastating disease, detection of which at early stages is important for better prognosis and increased life span of life. Conventional methods employed to date are mostly invasive and painful for the patients. Aggressiveness of these conventional invasive methods for diagnosis and prognosis of tumor calls for a dire need of less invasive, patient compliant, and reliable methods to diagnose the disease. The present study reviewed molecular and genetic biomarkers that can be employed for GBM cells, the study enlisted biomarkers that are of clinical use and also revealed the availability of just few biomarkers in last decade with promising results even after extensive research in GBM field. This condition indicates the aggressive nature of GBM cells and a need of extensive in this field. Endothelial growth factor receptor (EGFR), isocitrate dehydrogenase (IDH), tumor protein p53, loss of heterozygosity 10q, platelet-derived growth factor receptor alpha (PDGFRA), and circulating tumor cells acts as prognostic biomarkers where the concentration of these biomarkers act as disease progression or the success of therapy.

Though discovery of biomarkers and their employment in diagnosis and treatment is a tiring and strenuous journey, but the research would have to be done to find efficient techniques to combat the disease. This article would open the door to novel ideas for discovery of novel biomarkers and would provide a new insight to better incorporate already existing biomarkers for clinical use as there is urgent need to use the already known biomarkers in clinical practice based on patient specific biology. Moreover, biomarkers driven therapies, diagnosis, and prognosis would bring improvement in tumor's patient management and recovery.

Sarmad Sheraz Jadoon^1^, Umair Ilyas^2^, Hajra Zafar^3^, Ana Cláudia Paiva-Santos^4,5^, Saifullah khan^6^, Tanzeel Ahmed^7^, Yasir Rasool^8^, Reem Altaf^9^^∗^, Faisal Raza^3^∗, Muhammad Abbas^2^^∗^.

## Figures and Tables

**Table 1:** Summarized form of molecular biomarkers of glioblastoma multiforme.

Molecular biomarker	Importance	Source and examination	Functional significance	Regulation and prevalence rate
Endothelial growth factor receptor (EGFR)	Prognostic biomarker	Source: biopsy of GBM tissue.	Augmentation and mutation of EGFR alter tumorous cells of GBM through RTK/RAS/PI3K, SOX9, or FOXG1 pathways.	Amplification of EGFR.
		Examination: analysis of transfected cells or polymerase chain reaction		EGFRvIII (altered form of EGFR) is found along with wild-type EGFR.
				Commonly present in primary and classical subtype of GBM (40-50%)

Isocitrate dehydrogenase (IDH)	Prognostic biomarker	Source: biopsy of GBM tissue.	Alteration of IDH produces oncometabolite, 2-hydroxyglutarate (2-HG), which leads to the hypermethylation of DNA. As a result tumor genesis occurs.	Alteration of IDH1 and IDH2 in diffuse brain gliomas.
		Examination: analysis of transfected cells or polymerase chain reaction		Generally present in secondary GBM (85%) and in proneural subtype of GBM.

Tumor protein p53	Prognostic biomarker	Source: biopsy of GBM tissue	Increase tumor genesis by controlling isoprenoid or mavelonate pathway. Inactivated and degraded by MDM2.	Upregulation.
				Alteration in Tp53 gene is mainly present in secondary glioblastomas (90%) and in proneural subtype (67%) of GBM.

Methyl guanine methylene transferase	Predictive and prognostic biomarker	Source: biopsy by taking sample of non-necrotic GBM tissue	MGMT promoter after methylation gives improved prognostic results by using combination therapy (chemotherapy with TMZ adjuvant and radiotherapy) as compared to nonmethylated MGMT promoter.	Upregulation
		Examination: SYBR green technology and PCR with pyrosequencing		Present in both primary (64%) and secondary GBM (25%).

Loss of heterozygosity 10q	Prognostic biomarker	Source: biopsy by taking sample GBM tissue	It causes removal of tumor suppressor genes such as Tp53, NF1, and PTEN.	Upregulation
		Examination: magnification by PCR and consumption of microsatellites		It comprises major portion of GBM (70%) and mainly present in primary GBM (LOH10q23)

Circulating tumor cells	Prognostic biomarker	Source: body fluids such as blood	It helps to differentiate different molecular subclasses of GBM.	Upregulation
		Examination: telomerase assay.		It accounts for major portion of GBM (70%)
Platelet-derived growth factor receptor alpha (*PDGFRA*)	Prognostic biomarker	Source: biopsy by taking sample GBM tissue	Augmentation and alteration of PDGFRA contribute towards the GBM treatment	Found in secondary GBM and in proneural subtype of GBM
		Examination: polymerase chain reaction		

**Table 2:** Advantages and disadvantages of CTCs as biomarkers.

Pros and cons of tumor cells as biomarkers		
Circulating tumor cells	Provides information at DNA, RNA, or translated form of DNA (protein) level	Rarity (CTCs are less in number)
	Can perform functional assay	Presents challenging isolation technique

Circulating tumor DNA	ctDNA has better correlation with the stage of the disease	ctDNA has short half life
	ctDNA number in blood is more than CTCs	It is shed into the blood mostly by apoptotic or necrotic cells

Exosomes	Exosomes are easy to detect	They lack specificity, exosomes are not only removed by tumor cells but by all body cells
	They can carry DNA, RNA, and proteins	Exosomes may get contaminated during isolation process

**Table 3:** Summary of microRNAs being employed as biomarkers.

Biomarkers	Source	Importance of biomarker	Prevalence in glioblastoma and regulation	Functional importance	Reference
miR-21	Body fluids (blood, CSF, and urine)	Analytical and prognostic biomarker	Upregulation of miR-21 occurs	Modulation of certain genes for glioblastoma cells proliferation	[79]
miR-10b	Body fluids (blood, CSF, and urine)	Analytical and prognostic biomarker	Upregulation of miR-10b occurs	Induces Bcl-2 pathway inhibition and excessive proliferation of tumor cells	[81]
miR-181d	Body fluids (blood, CSF, and urine)	Analytical and prognostic biomarker	Downregulation of miR-181d occurs	miR-181d has inverse correlation with MGMT expression	[82]
miR-137	Body fluids (blood, CSF, and urine)	Analytical and prognostic biomarker	miR-137 is downregulated	miR-137 has negative regulation effect on its gene target GLIPR-1	[82]
miR-15b	Body fluids (blood, CSF, and urine)	Analytical and prognostic biomarker	MiR-15b is downregulated	miR-15b inhibits cell cycle progression in normal cells while in GBM, it is downregulated, hence inducing cell cycle progression	[83]

**Table 4:** lncRNA as therapeutic targets in glioblastomas.

LncRNA	Importance of biomarker	Functional importance	Reference
CASC7	Reduces glioma formation and progression	Acts by reducing the wnt/b-catenin activity, thereby reducing the glioma formation and progression	[86]
CASC9	Promotes glioma formation and progression	This lncRNA along with miR-519d and STAT3 promotes the glioma formation and progression by forming a positive feedback loop	[87]
AGAP2-AS1	Promotes glioma formation and progression	This lncRNA also acts by activating wnt/b-catenin pathway resulting in glioma formation	[88]
NEAT1	Promotes glioma formation and progression	Interacts with polycomb repressive complex subunit EZH2 thought he wnt/b-catenin pathway causes tumor formation and tumirogenesis	[89]
LINC01426	Promotes glioma formation and progression	Initiates the glioma initiation by acting through P13K/Akt signaling pathway	[90]
PART1	Tumor suppressor lncRNA	Downregulating the PTEN/Akt signaling pathway through sponging miR-190a-3p	[91]
LINC01446	Promotes tumor progression	Acts through miR-489-3p/TPT1 axis	[92]
MNX1-AS1	Promotes glioblastoma progression	Acts by inhibiting miR-4443	[93]
DCST1-AS1	Promotes proliferation of tumor	Acts by decreasing mir-29b levels through methylation	[94]
AC016405.3	Tumor suppressor	Causes TET2 modulation by acting as molecular sponge for miR-19a-5p.	[95]
HOTAIRM1	Promotes tumor malignancy	Facilitates interaction of long-range chromatin interactions with HOXA genes resulting in increased transcription	[96]
HOXB13-AS1	Promotes tumor progression	Regulates HOX gene transcription	[97]
LINC00467	Promotes tumor progression	Decreases the tumor suppressor p53 by interacting with DNMT1	[98]
HIFiA-AS2	Promotes mesenchymal tumors	Maintains mesenchymal GSCs in hypoxic niches	[84]
H19	Promotes glioma invasion	Promotes glioma invasion in HIF-1a dependent manner	[99]
LINC01494	Promotes tumor migration	Titrate wit miR-122-5p causing increased CCNG1 expression	[100]
ATB	Promotes glioma cell invasion	Acts through NF-*κ*b and MAPK signaling pathways.	[101]
GAS5	Suppress tumor invasion and survival	Targets GSTM3 expression	[102]
Lnc-TALC	Promotes resistance to TMZ and tumor recurrence	Regulates the c-met pathway and promotes the O6-methylguanine-DNA methyltransferase (MGMT) expression	[103]
MALAT1	Promotes TMZ resistance and invasion	Acts by restoring p53 activity and expression	[104]
ADAMTs9-AS2	Promotes TMZ resistance	Changes the ubiquitination mediated by FUS/MDM2	[105]
TP73-AS1	Promotes TMZ resistance and metabolism in GSCs	Regulates the GSC/therapy resistance marker ALDH1A1	[106]
NCK1-AS1	Increases TMZ resistance	Acts through disinhibition of TRIM24	[107]
HMMR-AS1	Causes radiation resistance, tumor progression, and invasion	Acts by targeting ATM, RAD51, and BMI1	[108]
TALNEC2	Causes radiation resistance and promotes tumor progression	Regulates growth and stemness in glioma stem cells	[109]
PCAT1	Increase sensitivity to radiation	Acts by modifying HMGB1	[110]

**Table 5:** Genetic symbols along with involved pathways.

Genetic symbol	Pathways involved	Cancer events associated with particular gene	References
CTSZ	STAT5 JAK/IL2 pathway	Persistent signals of cell division, activation of invasive, and metastatic pathway	[114]
EFEMP2	Transition of epithelial mesenchymal cells		
SOCS3	Response to interferon gamma	Sustained proliferative signals, inflammation associated with tumor spread	[115]
	NF-*κ*B signaling pathway, IL6/JAK/STAT3 signaling		
SERPINE1	Transition of epithelial mesenchymal cells	New blood vessels formation	[114]
	TNF- *α* signaling		
	TGF-*β* signaling,		
PLAUR	TNF- *α* signaling	Activation of invasive and metastatic pathway	[114]
	Cholesterol maintenance	New blood vessels formation	
MAP 2 K3	TNF-*α* signaling	Sustained signals of cell division	[114]
	mTORC1 signaling		
	PI3K/AKT/mTOR signaling		
MICALL2			[114]
MDK	Apical junction		[114]
	Estrogen response late		
KDELR2		Activates metastatic pathway and invasion	[114]
ITGA5	Epithelial mesenchymal transition, and inflammatory response		[114]
